# Editorial for the Special Issue “Healthcare-Associated Infections and Antimicrobial Therapy—2nd Edition”

**DOI:** 10.3390/microorganisms14020267

**Published:** 2026-01-23

**Authors:** Petros Ioannou, Diamantis Kofteridis

**Affiliations:** 1 School of Medicine, University of Crete, PC 71003 Heraklion, Greece; 2 Internal Medicine Department, University Hospital of Heraklion, PC 71110 Heraklion, Greece

Healthcare-associated infections (HAIs) remain a major cause of morbidity, mortality, and healthcare cost worldwide, posing one of the most critical challenges in medicine nowadays [1,2,3]. The global spread of multidrug-resistant (MDR) and extensively drug-resistant (XDR) organisms, coupled with limited novel antimicrobial options, has increased the urgency for coordinated research, effective infection control, and antimicrobial stewardship [4,5,6,7]. To that end, research on healthcare-associated infections and antimicrobial therapy is of paramount importance, to allow better understanding of the evolving concepts of antimicrobial resistance and its implications in everyday clinical care.

This Special Issue of Microorganisms, “Healthcare-Associated Infections and Antimicrobial Therapy—2nd Edition,” continued the mission of its predecessor by gathering original research and reviews that address the epidemiology, diagnosis, prevention, and treatment of HAIs across diverse clinical settings. It is free and available at the following link (https://www.mdpi.com/journal/microorganisms/special_issues/01AP3IHN01, (accessed on 15 January 2026)) and encompasses eight articles that address the abovementioned questions.

More specifically, Tsolakidou et al. conducted a comprehensive two-year analysis of blood culture data in a secondary hospital in Western Greece. Their study evaluated contamination rates, pathogen profiles, and antimicrobial susceptibility, highlighting the predominance of Gram-negative pathogens such as *Escherichia coli* and *Klebsiella pneumoniae*, alongside notable resistance patterns. The findings underscore the importance of continuous microbiological surveillance and quality control in laboratory diagnostics. By identifying contamination sources and pathogen trends, the study contributes to refining infection control policies and optimizing empirical therapy in resource-limited healthcare environments.

Bukavaz et al. introduced a rapid, reliable SYBR-Green-based polymerase chain reaction (PCR) assay capable of detecting major carbapenemase genes—blaKPC, blaNDM-1, and blaOXA-48—in *K. pneumoniae* and *Acinetobacter baumannii*. The assay’s ability to deliver results within 65 min marks a significant advance in diagnostic speed, enabling earlier targeted therapy and infection control measures. The study demonstrates how simplified molecular platforms can complement routine microbiology in the early detection of resistance, a cornerstone for controlling the spread of carbapenem-resistant organisms in clinical settings.

Boyanova et al. explored the genetic diversity and resistance patterns of 73 *Cutibacterium acnes* strains isolated from acneic and non-acneic patients. Using phylotyping and susceptibility testing, they identified a predominance of type IA1 and increasing resistance to macrolides and clindamycin. The study highlights that even commensal organisms can serve as reservoirs of resistance genes and that antibiotic misuse in dermatology contributes to resistance spread. Their data emphasize the need for region-specific surveillance and antimicrobial stewardship in skin-related infections.

Alshammari and Alruwaili presented an important epidemiological analysis of HAIs among intensive care unit (ICU) patients in Aljouf, Saudi Arabia. Their retrospective study identified a significant prevalence of ventilator-associated pneumonia and bloodstream infections, primarily due to *Acinetobacter baumannii* and *Pseudomonas aeruginosa*. Identified risk factors included prolonged hospitalization, mechanical ventilation, and prior antibiotic exposure. The authors advocate for strengthening infection prevention programs and implementing regional antimicrobial stewardship strategies to mitigate the burden of HAIs in critical care settings.

De Capitani et al. examined clinical outcomes in patients with catheter-related and catheter-associated bloodstream infections (CRBSI/CABSI), comparing catheter removal versus retention strategies. Their single-center retrospective analysis revealed that early catheter removal correlated with improved outcomes, particularly in infections caused by Gram-negative pathogens or *Candida* species. However, selective retention could be safe under specific microbiological and clinical conditions. The study provides practical evidence guiding individualized catheter management and underscores the importance of timely intervention in bloodstream infections.

Voinescu et al. performed a comparative evaluation of bacterial conjunctivitis across adult and pediatric cohorts in inpatient and outpatient settings. Their findings revealed distinct pathogen distributions—*Staphylococcus aureus* predominated in adults, whereas *Haemophilus influenzae* was very common in children. Resistance rates to fluoroquinolones and macrolides varied significantly between groups, suggesting the need for age- and context-specific therapeutic guidelines. The study adds valuable epidemiological insight into ophthalmic infections and their evolving resistance trends in hospital versus community contexts.

Gravante et al. reviewed contamination risks in Starting Active Materials for Synthesis (SAMS) and their implications for drug manufacturing and public health. The review synthesizes international regulatory frameworks governing microbial contamination control, emphasizing the intersection between Good Manufacturing Practices (GMP) and infection prevention. The authors highlight the need for harmonized microbiological testing and risk assessment to prevent contamination-related product recalls or patient harm. This work bridges industrial microbiology with healthcare safety, providing a regulatory perspective on antimicrobial control beyond clinical settings.

Ioannou et al. provided a detailed synthesis of human infections caused by *Kytococcus* spp., a rare but clinically relevant genus of Gram-positive cocci. The review compiles case reports and clinical data revealing *Kytococcus schroeteri* as the predominant species implicated in prosthetic and endocardial infections. The authors discuss diagnostic challenges and antimicrobial susceptibility profiles, noting resistance to beta-lactams and variable susceptibility to glycopeptides. This comprehensive overview raises awareness of these under-recognized pathogens, underscoring the need for accurate identification and tailored therapy.

Collectively, the eight contributions in this Special Issue highlight the dynamic landscape of healthcare-associated infections and the persistent challenge of antimicrobial resistance. From laboratory innovation and clinical epidemiology to regulatory oversight and emerging pathogens, these studies illustrate the necessity for an integrated, multidisciplinary approach to infection control. Future research should aim to refine rapid diagnostic tools, expand stewardship programs, and explore novel therapeutic combinations against resistant organisms. Continued international collaboration remains essential to preserve antimicrobial efficacy and safeguard global public health.
